# Recent Advances of Hepatitis B Detection towards Paper-Based Analytical Devices

**DOI:** 10.1155/2021/6643573

**Published:** 2021-02-26

**Authors:** Aulia A. Tyas, Septi F. Raeni, Setyawan P. Sakti, Akhmad Sabarudin

**Affiliations:** ^1^Department of Chemistry, Faculty of Science, Mahidol University, Bangkok 10400, Thailand; ^2^Departement of Chemistry, Faculty of Science, Brawijaya University, Malang 65145, Indonesia; ^3^Department of Physics, Faculty of Science, Brawijaya University, Malang 65145, Indonesia; ^4^Research Center for Advanced System and Material Technology, Brawijaya University, Malang 65145, Indonesia

## Abstract

Hepatitis B virus (HBV) still remains a major global public health problem. One-half to one-third of the total HBV infected people died due to late detection of HBV. Serological antigen and viral HBV detections can help in the diagnosis, referral, and treatment of HBV. Available methods for HBV detection mostly used bulky instruments. Miniaturization of devices for HBV detection has been started by narrowing down the size of the devices. Several methods have also been proposed to increase the selectivity and sensitivity of the miniaturized methods, such as sandwich recognition of the biomarkers and the use of nano- to micro-sized materials. This review presents recent HBV detections in the last two decades from laboratory-based instruments towards microfluidic paper-based analytical devices (*µ*PADs) for point-of-care testing (POCT) purposes. Early and routine analysis to detect HBV as early as possible could be achieved by POCT, especially for areas with limited access to a central laboratory and/or medical facilities.

## 1. Introduction

Hepatitis B virus (HBV) is known as a type of hepatitis that can cause acute and chronic liver diseases such as cirrhosis and hepatocellular carcinoma. Recently, in every two billion HBV infected cases, approximately 20% of them continue to chronic stages, and it causes nearly 900,000 deaths every year. Determination of the stage or phase of infection is necessary to guide decisions in giving antiviral therapy. Individual infected HBV symptoms are darker color urine, jaundice, yellowing of the skin and eyes, abdominal pain, jaundice, vomiting, and nausea depending on individual immunity. Even though the average prevalence rate decreases every year and HBV vaccines are currently available, HBV is still a major health problem worldwide. It is because of its easiness transmission through sexual contact and body fluids contact, especially blood. The World Health Organization (WHO) recommends people must be tested for HBV before blood donation and transfusion, liver transplantation, and giving birth processes [1–3]. Thus, early diagnosis of HBV infection is required to prevent transmission and long-term complications and reduce death cases.

Several HBV biomarkers can be used for HBV diagnosis such as hepatitis B surface antigen (HBsAg), hepatitis B surface antibody (anti-HB), hepatitis B e-antigen (HBeAg), and hepatitis core antigen (anti-HBc). The quantification of biomarkers level in the body fluids determines the infection level of HBV. Classical methods of HBV detection, e.g., polymerase chain reaction (PCR), enzyme-linked immunosorbent assays (ELISA), radioimmunoassay (RIA), and enzyme immunoassay (EIA), as well as newer technologies such as electrochemiluminescence immunoassay (ECLIA), microparticle enzyme immunoassay (MEIA), and chemiluminescent microparticle immunoassay (CMIA) have been used widely for many years owing to its robustness and high accuracy. Immunoreactions between antigens and antibodies linked to enzymatic reactions in a serological sample are the basis for ELISA's screening and diagnosis. HBV DNA can also provide information about the presence of strange DNA strands, determination of the infectious risk, treatment decision, and monitoring infection [3–5]. The aforementioned methods for serological and viral testing of HBV are often expensive and require bulk instrumentation and laboratory personnel training. However, high HBV countries are often low-to middle-income countries that cannot afford or have limited access to such specialized instruments.

Over the last two decades, researchers have developed other methods employing advanced technologies to improve HBV detection in many ways. The most common one is hepatitis B biosensors. Hepatitis B biosensors are detection devices that use biological matters to detect the presence of HBV biomarkers in the biological body fluids with various detection systems such as colorimetric [6], fluorescence [7], Raman spectroscopy [8], and electrochemical detections [9, 10]. These developed methods are showing potential for HBV biomarkers detection.

Point-of-care testing (POCT) is described as alternative testing prepared close to the patients who have difficulty accessing diagnostic laboratories [11, 12]. In general, the development of POCT devices has been hindered by technical challenges in scaling down, automating, and integrating all testing steps such as sample pretreatment, amplification (if needed), detection, and interpretation result using a single “sample-to-answer” device. Therefore, research on the development of POCT devices has been focused on miniaturizing and improving the performance of the devices [13]. It would be more beneficial if the analytical devices could be used by whomever in need. The microfluidic paper-based analytical devices (*µ*PADs) from the first proposal have changed to many scaled-down analytical devices, including *µ*PADs to detect HBV [12]. This review paper presents the transformation of HBV detections in the last two decades. The transformation aimed to provide alternative methods to detect HBV to be more accurate, rapid, robust, and user-friendly and less waste-producing and more affordable and biodegradable devices leading to POCT devices.

**Figure 1:**
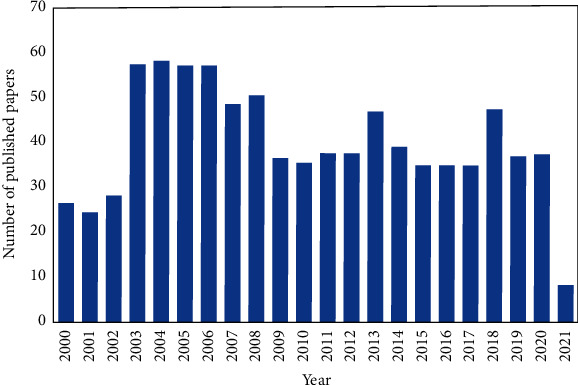
Number of published papers dealing with detection/determination of hepatitis B virus in the last 2 decades (source: scopus.com, accessed on February 18, 2021).

According to scopus.com, in the last 2 decades (2000–2021), the number of papers published dealing with the detection or determination of hepatitis B virus was 858 articles, as shown in Figure 1. However, the articles discussing the potential point of care testing (POCT) method such as paper-based analytical devices (PADs) for detecting hepatitis B were still very few, which is only about 5 articles published since 2015. Therefore, research on POCT to detect hepatitis B virus still provides a tremendous opportunity for further development. Additionally, this review is important as a trigger to other researchers to develop POCT using PADs platform because it is affordable, easy-to-use, and suitable in the most developing countries.

## 2. Common Methods for Detection of Hepatitis B Virus

### 2.1. DNA Amplification Method

Polymerase chain reaction (PCR) is well-known as a sensitive technique for detecting HBV DNA in biological samples. However, conventional PCR requires high cost and long analysis time. In recent years, to shorten the analysis time, reduce the analysis cost, and increase PCR sensitivity, researchers have modified the existing PCR technique. For this purpose, a single-step triplex PCR assay to detect all HBV genotypes was developed [14]. Utilizing *β*-actin as the internal control genes to check the quality of samples, this developed PCR technique could identify the condition of samples that may deteriorate during transportation, especially in rural areas. The sample condition is important to determine the reliability of the analysis. A coamplification at lower denaturation temperature PCR (COLD-PCR) coupled with probe-based fluorescence melting curve analysis (FMCA) to quantify HBV DNA, genotype, and mutation was also reported [15]. This work presents high sensitivity despite the use of lower denaturation temperature on PCR.

Unlike PCR, which needs operations at different temperatures in each step, a loop-mediated isothermal amplification assay (LAMP) only requires a constant temperature for reaction and amplification steps. Closed tube LAMP assay for fluorescence detection of HBV DNA was employed to prevent cross-contamination [16]. In this method, 6 HBU primers were required to identify the target DNA sequence regions specifically, allowing the amplification time to become shorter (less than half an hour) than the PCR technique.

### 2.2. ELISA-Based Colorimetric Detection

Colorimetry is a detection method based on the reaction that results in the color change or color intensity. The advantages of colorimetric detection are easy operation, instrument-free (could be seen by naked eyes), and relatively low cost. The occurred reaction must be specific to detect the target analyte. HBsAg is often chosen as the common biomarker to show the presence of HBV in human serum. Immunoreactions are usually employed to detect HBsAg specifically. Enzyme-linked immunosorbent assay (ELISA) is the well-known common method to detect or quantify biological molecules' presence with specific interaction antigens against respective antibodies. ELISA-based colorimetric detections have been developed to detect and determine the amount of HBsAg by various recognition mechanisms. A direct sandwich ELISA to detect HBsAg was developed by Kim in 2017 [6]. In this method, the optimized monoclonal antibodies (mAbs) to capture and horseradish peroxidase (HRP) enzyme linked-antibody were H17 and H31 mAbs, respectively. The direct sandwich ELISA is used to increase the specificity of HBsAg recognition by utilizing two antibody recognition systems. A 3,3′,5,5′-tetramethylbenzidine substrate was added to quantify the amount of captured HBsAg. The optical density obtained at 450 nm was proportional to the concentration of HBsAg. An indirect sandwich ELISA system was developed by Yucel and Akcael in 2018 [17]. They were using 2G3 mAb as the capturing agent and polyclonal antibodies-biotin as the detecting agent. The comparability of the obtained ELISA results compared to that from commercial ELISA kits was 97%. Analytical performances of the colorimetric detection of HBV are listed in Table 1.

### 2.3. Fluorescence Detection Method

Fluorescence is a signal transducer measuring the emitted photons as the results from molecules absorbing light. Usually, the emitted photon has a longer wavelength than the excitation light [18]. A fluorescence enhancement strategy based on silver nanoparticle (AgNP) aggregates for detecting HBV DNA sequences was developed through functionalization of AgNPs with recognition probes (Cy3-probe) and hybrid probes (oligomers). The presence of target DNA mediated the formation of sandwich complexes between the immobilized capture probes and the functionalized AgNPs, which was followed by the hybridization-induced formation of AgNP aggregates. The fluorescent intensity could be enormously amplified by the increasing number of fluorophores and metal enhanced fluorescence (MEF) effect. Moreover, this method had high specificity for distinguishing single-base mismatches and identifying target DNA under genomic DNA interference. This method also performed a reasonable specificity for distinguishing the perfect matched sequences from the single-mismatched sequences. This fluorescent microarray based on AgNP aggregation had high-throughput analytical potential. It provided a fast and easy approach to detect HBV DNA sequences with a wide linear range over five orders of magnitude. With further refinement, it will be used for multiplexed disease diagnoses [19].

The surface-modified europium nanoparticles (EuNPs) as a fluorescent probe to detect the presence of HBsAg in serum samples was also reported [20]. The goat anti-HBsAg pAb was chemically biotinylated with biotin isothiocyanate to form bio-pAb. HBsAg mAb was also covalently bound on the surface of EuNPs. Bio-pAb was then immobilized in a streptavidin-coated plate followed by EuNP-mAb. The modified plate was then left to dry, called a dry-reagent immunoassay. A buffered sample solution was loaded in a well of the dry-reagent assay to analyze a sample and measured the fluorescence intensity. Another sensitive and straightforward Sandwich fluorescence ELISA for detecting HBsAg was reported by Wu et al. [21]. Anti-HBsAg mAb was used as the captured antibody, and biotinylated-glucose oxidase (biotin-GOx) was used as enzyme-linked pAb. The fluorochrome used was mercaptopropionic acid-modified CdTe quantum dots (MPA-QDs). In the presence of HBsAg, biotin-GOx could catalyze glucose to produce gluconic acid and H_2_O_2_. The produced H_2_O_2_ in the well could quench the fluorescence signal of MPA-QDs. Moreover, the proposed assay exhibited good accuracy and reproducibility. It could be comparable with TRFIA in reliability for HBsAg quantification. Like another ELISA technique, this method produced high sensitivity and selectivity that could serve as a potential platform for the rapid and highly sensitive detection of biomarkers in clinical diagnosis.

For routine analysis, a portable device to detect HBsAg in human serum is an immunochromatographic assay (ICA) for detection of HBsAg which was presented by Shen et al. [22]. Carboxyl-modified quantum dot beads (QBs) were employed as the fluorescent probe. The QBs were then labeled with anti-HBsAg mAb forming QB-mAb. The QB-based ICA platform consisted of 3 parts: sample pad, nitrocellulose (NC) membrane, and absorbent pad. On the NC membrane, goat polyclonal antibodies (G-pAbs) and donkey polyclonal antibodies (D-pAbs) were immobilized on the test (*T*) and control (C) lines, respectively. The detection principle of the QB-based ICA is based on sandwich immunoassay. The serum sample was mixed with QB-mAb probe and loaded on the sample pad. Once the HBsAg is present in the sample, it would bind with QB-mAb to form QB-mAb-HBsAg complex. The complex could bind with G-pAbs and D-pAbs, resulting in two fluorescent signals on *T* and C lines. On the other hand, the absence of HBsAg would result from one line, on C line only. For quantitative analysis, both lines' fluorescence intensity was directly proportional to the concentration of HBsAg [22].

Hyperbranched rolling circle amplification- (HRCA-) based fluorescence for detection of HBV single-base mutant DNA (mutDNA). HRCA consists of three steps; firstly, the mutDNA was designed to be circular by connecting 5′ and 3′ ends with the help of DNA ligase, called padlock probe. After removing the noncircularized probe, the HRCA reaction occurred with help from Bst DNA polymerase, primer 1, primer 2, and dNTPs to form double-stranded DNA (dsDNA) and single-stranded DNA (ssDNA). This reaction is particular to mutDNA as the HBV target DNA. Even though, there was only one base difference in the DNA sequence, the padlock probe cannot be formed. Lastly, the addition of SYBR green I, which could be inserted in dsDNA with high affinity, gave a very strong fluorescence signal [7]. The HRCA is a powerful amplification technique resulting in highly specific and selective quantification of HBV DNA. Analytical performances of the fluorescence detection of HBV are listed in Table 2.

### 2.4. Raman Spectroscopic Method

Raman spectra measure the scattered light from a vibrational molecule; therefore, this method can identify the combination of a chemical structure and target analyte. A fluorescent molecule can interfere with the measurement of Raman spectra due to its emission at a longer wavelength. Tong et al. in 2019 have demonstrated an adaptive iterative weighted penalty least squares method (airPLS) to minimize the fluorescence spectral interference making the Raman efficiency higher. The airPLS was coupled with a principal component analysis (PCA), particle swarm optimization (PSO) algorithm, and support vector machine (SVM) to build an airPLS-PCA-PSO-SVM Raman spectroscopy. The algorithms successfully eliminated fluorescence spectral interference and improved the data processing to get the exact results of Raman spectra. The serum samples containing HBsAg and HBV DNA were prepared the same way as PCR analysis. The percentages of accuracy, specificity, and sensitivity of the proposed Raman spectroscopy built with the airPLS-PCA-PSO-SVM were 93.1%, 80%, and 100% [8].

Rather than the improvement of the software, Kamińska et al. have developed a surface-enhanced Raman spectroscopy (SERS) to determine the concentration of HBsAg in human blood plasma. Glass slides are usually used as SERS substrate for immunoassay-based Raman spectroscopy, but it is not SERS-active substrate. In this work, they developed an Au–Ag coated on GaN to prepare the SERS-active substrate. The surface enhancement factor increased by million times with high reproducibility and stability. The SERS-active surface was modified with 6-amino-1-hexanethiol to make the amino-terminated linkage. The linkage was useful to attach mAb of anti-HBsAg to the SERS-active surface. The detection principle of this SERS-active substrate follows the one of sandwich ELISA, so another anti-HBsAg mAb was labeled with gold nanoflowers (AuNFs) and fuchsin (FC) as Raman reporter forming anti-HBsAg/AuNFs/FC. The introduction of all the reagents was performed using a microfluidic chip made of polycarbonate integrated with the prepared SERS-active substrate. In the presence of HBsAg, the Raman shift signals increased proportionally to the concentration of HBsAg [23].

Similar to the SERS-active substrate, a single-layered localized surface plasmon resonance (LSPR) sensing technique is based on the measurement of the resonance condition of a localized surface, such as gold nanoparticles (AuNPs), due to the incidence of light sending on to the surface of AuNPs. The incident light will be reflected at a certain angle generating the surface plasmon. If the metal surface composition differs from that of the original metal, the resonance condition changes. This phenomenon could be used to detect and quantify macromolecules such as HBsAg attached to the surface of a metal. An antigen-HBsAg was immobilized on the surface of a glass substrate followed by incubation of HBsAg. Another anti-HBsAg conjugated AuNPs (immunocolloidal gold) was attached to the HBsAg, and the wavelength shift was determined as the recorded signal. The higher complex amount attached to the surface of the glass substrate caused the longer wavelength shifting [24].  Table 3 lists the analytical performances of Raman spectroscopy detection of HBV.

### 2.5. Electrochemical Detection Method

Electrochemical detections of HBV biomarkers are potentiometry, amperometry, and voltammetry. The analytical performances of each method are summarized in Table 4.

#### 2.5.1. Potentiometry

Potentiometry immunoassay based on the change in potential response (in the absence of current) of an assay depends on the immunoreaction. To selectively detect the desired molecule, modification of the working electrode is essential. Several characterization techniques are used to confirm whether the modification is successfully made, such as cyclic voltammetry (CV) and electrochemical impedance spectroscopy (EIS). A (3-mercaptopropyl) trimethoxysilane (MPS) sol-gel modified gold electrode was immersed in the prepared AuNP solution following by incubation in anti-HBsAg. AuNPs in the working electrode allows anti-HBsAg to bind on the MPS sol-gel modified gold electrode covalently. The potentiometric response measured against standard calomel electrode (SCE) corresponds to the concentration of HBsAg [10]. Potentiometric measurement of HBsAg utilizing Sandwich ELISA's principle has improved the selectivity of the analytical method. The precoated mAb anti-HBsAg in the microtiter plate recognized HBsAg. Since HBsAg was present in the microtiter plate, Ru(bpy)_3_^2+^-NHS ester labeled pAb anti-HBsAg could bind with HBsAg. The immunocomplex was desorbed from microtiter using NaOH. The elution in PBS (pH 8.0) as running buffer measured the potentiometric responses using capillary electrophoresis-electrochemiluminescence [25].

#### 2.5.2. Amperometry

An amperometric measurement is a measurement of current as a result of an applied potential through the system. Conventional electrochemistry methods are operated using 3D electrodes. Carbon, glassy carbon, gold, and platinum electrodes are commonly used as the working and counter electrodes, while Ag/AgCl is the reference electrode. A sandwich electrochemical immunosensor was built from a modified glassy carbon electrode (GCE) with gold nanoparticles/polypyrrole nanosheet (AuNPs/PPy NS). The AuNPs/PPy NS/GCE was incubated in anti-HBsAg (Ab_1_) to immobilize Ab_1_. The second anti-HBsAg (Ab_2_) was labeled with Rh as core and Pt as shell nanodendrites through amino linkage to graphene nanosheet (RhPt NDs/NH_2_-GS/Ab_2_). The current response was measured at applied potential −0.4 V over time. As the concentration of HBsAg increases, the number of sandwich immunocomplex molecules formed on the AuNPs/PPy NS/GCE increases, decreasing amperometric signal response [26]. This condition could be a disadvantage if the concentration of HBsAg is too much; there will be current flows as the immunocomplex molecules hinder the electron transfer process. Therefore, there have been proposed PdCu tripod alloys loaded in porous graphene (PdCu TPs/PG) immobilized on a GCE, which was incubated in peroxidase nanoenzyme. Then, PdCu TPs/PG/GCE was incubated in anti-HBe. HBeAg could be detected without labeling. The signal responses were measured over time using chronoamperometry mode [27]. It has been proven that the signal enhancement was achieved due to loading PdCu TPs in the PG, leading to increasing electrochemical active area.

The 2D amperometric immunosensor integrated with near field communication enabling a wireless operation device also has been proposed. The screen-printed graphene electrode (SPGE) was modified with AuNPs linked to *β*-cyclodextrin to enhance the signal and improve the selectivity to determine HBsAg. The modification was done by electrodeposition of AuNPs onto SPGE and electropolymerization of *β*-cyclodextrin onto AuNPs-SPGE. Anti-HBsAg was capped in the *β*-cyclodextrin, and HBsAg was directly measured without tagging. The amperometric signal was recorded in the absence and the presence of HBsAg [28]. The proposed device has shown a promising portable device for rapid and reliable detection of HBsAg with satisfying performance.

#### 2.5.3. Voltammetry

Voltammetry is a technique measuring current flows in an electrochemical cell by controlling the applied voltage. There are several types of voltammetry, and the most common one is cyclic voltammetry, which can be used to qualitatively and quantitatively measure electrochemical properties of an electrochemical cell or a chemical species [35]. Electrochemical immunosensors have been developed to determine HBV biomarkers utilizing cyclic voltammetry (CV), square-wave voltammetry (SWV), and differential pulse voltammetry (DPV) as signal transducers. An electrochemical sandwich-type immunosensor was performed in a container. Anti-HBsAg-conjugated magnetic nanoparticles (anti-HBsAg-MNPs) were immobilized in the container employing magnetic properties of streptavidin-coated nanoparticles. Horseradish peroxidase-conjugated with another anti-HBsAg (HRP-anti-HBsAg) would attach in the presence of HBsAg to form immunocomplex molecules. Aminophenol as the substrate of HRP measured the electrochemical response in CV mode using the 3D three-electrode system with GCE against Ag/AgCl as the working and reference electrodes. Increasing current responses of cathodic peak represented the increase of HBsAg concentration [29]. The use of another voltammetry mode, such as SWV and DPV, is aimed to improve the sensitivity of the measurement.

A dual amplified electrochemical immunosensor has been developed in the same year. GCE was coated with graphene oxide (GO) and chitosan followed by a coating of anti-HBsAg 1 (Ab_1_) to get GCE/GO/Ab_1_. In this work, there are two proposed immunosensors, one GCE/GO/Ab_1_ was incubated with prepared hemin/G-quadruplex conjugated Fe_3_O_4_–Au nanocomposites (Ab_2_/Fe_3_O_4_-AuNPs/hemin-G-quadruplex/MB) and the other one was incubated with prepared Ab_2_/(H-amino-rGO-Au) nanohybrid/hemin-G-quadruplex/MB. The hemin-G-quadruplex is one of the synthetic DNAs like an enzyme (DNAzyme). The function of DNAzyme is to catalyze hydrogen peroxide in the presence of methylene blue (MB) as a mediator [32]. A high sensitivity of sandwich immunosensor has also been reported using gold nanorods (AuNRs) and mesoporous Au@Pd@Pt (m-Au@Pd@Pt). GCE surface was covered with AuNRs followed by immobilizing of anti-HBsAg 1 (Ab_1_). Ab_2_ was labeled with the prepared m-Au@Pd@Pt [33]. In this work, the detection principle is HBsAg as the target molecule will be bound with Ab_1_ and Ab_2_ to form a sandwich-like electrochemical immunoassay. The signal responses of those two proposed immunosensors were recorded using DPV.

Nowadays, many researchers are going into label-free detection of biomolecule instead of labeled detection methods. Label-free detection allows biomolecule determination to be faster and less reagent consumption as the preparation step is simpler than labeled detection. This method can lead to the rapid and real-time detection of HBsAg. A label-free immunosensor was made by growing polytyramine (PTy) film directly on the gold electrode (GE) surface using electropolymerization in an acidic medium. The GE was then incubated in the dispersed carbon nanotubes (CNTs), the surface of PTy film bonded with–COOH group of CNTs. Immobilization of antihepatitis B core antigen (anti-HBc) onto the surface of modified GE surface was done by immersing it in the anti-HBc solution to get PTy/COOH-CNT/anti-HBc/GE. The unbounded PTy film was then covered with glycine. The current response was recorded using SWV by immersing the 3D three-electrode system in HBcAg sample solution for 15 min. The prepared immunosensor could get the current signal without HBcAg labeling. It can produce faradaic signal from the use of PTy-CNT composite film due to the presence of derivative amine groups in the film [30]. There has also been reported a label-free immunosensor based on PtPd nanocubes coated on MoS_2_ (PtPd NCs@MoS_2_) by Tan et al. The prepared PtPd NCs@MoS_2_ was then coated on the GCE surface followed by immersing it in the anti-HBsAg solution. The current response in the presence of HBsAg was measured using the DPV mode. The PtPd NCs@MoS_2_ could generate high current responses, so labeling HBsAg is not necessary [31].

To date, aside from HBsAg, HBV DNA also has been used as the biomarker of HBV. A sensitive electrochemical DNA sensor was developed based on sandwich structure molecules utilizing new unique Cu_3_(PO_4_)_2_-BSA-GO nanoflower particles, AuNPs, and two surface-immobilized aptamers to amplify the signal. The nanoflowers were fabricated using a very simple one-step procedure and provide binding points for AuNPs. Two aptamers were designed with thiol and ferrocene, respectively, on the 5′-terminus and 3′-terminus to increase sensor performance. The complex biosensor layers provide sufficient binding points and an adaptive framework for signal amplification and biocompatibility. The excellent performance in HBV propagation detection suggests that the proposed electrochemical DNA (E-DNA) sensor has great application potential in clinical diagnosis. The present E-DNA sensor is simple, rapid, sensitive, specific, and affordable [2]. A genosensor mechanism to indirectly detect ssDNA of HBV has been developed as well. A graphite disk used as the working electrode was coated with poly(4-aminophenol) film by electropolymerization method. An ssDNA was then immobilized on the surface of the modified working electrode. Hybridization of the DNA probes was done at 42°C. Ethidium bromide was able to be inserted into the dsDNA for detection purposes [34]. Both E-DNA and genosensor were utilizing DPV to obtain the current responses.

### 2.6. Dynamic Light Scattering Method

Sandwich-type immunocomplex containing target molecule is usually detected through its optical or electrochemical properties. Wang et al. have demonstrated a new way to quantify HBsAg based on its immunological complex utilizing AuNPs' size of 50 to 100 nm. AuNPs-50 and AuNPs-100 were labeled with mAb and pAb of HBsAg, respectively. The immunological reaction took place after incubation; the hydrodynamic size of the immunocomplex was measured using a dynamic light scattering (DLS) instrument. The hydrodynamic size of the immunocomplex corresponded to the concentration of HBsAg as the more HBsAg could attach to the pAb epitope sites. The linearity and detection limit of the proposed method were 0.005–1 IU·mL^−1^ and 0.005 IU·mL^−1^, respectively [36].

### 2.7. Spectroelectrochemistry

#### 2.7.1. Electrochemical Impedance Spectroscopy

Electrochemical impedance spectroscopy (EIS) measures the total impedance of an electrochemical system by applying an AC voltage at various frequencies for detection purposes. An electrochemical circuit consists of resistance and capacitors in series or parallel configuration. In the equivalent electrical circuit of immunosensors, there are *R*_s_, solution resistance; *R*_ct_, charge transfer resistance; *C*, double-layer capacitance; *W*, Warburg impedance; and other constants. Electrochemical immunosensors based on EIS measurement mostly focus on the change in *R*_ct_ to identify the immunoreaction, which indicates the attachment of the target analyte on the electrode surface [9, 37, 38].

An immunosensor was fabricated from in situ single-walled carbon nanotubes (SWCNTs), then was electrochemically coated with AuNPs to be SWCNTs/AuNPs. The SWCNTs/AuNPs were incubated in an ssDNA probe to obtain SWCNTs/AuNPs/ssDNA working electrode. The hybridization of the target DNA on the working electrode surface was performed at 45°C for 2 h [9]. Another genosensor to determine HBV DNA was fabricated from electrochemical oxidation of aluminum to acquire anodic aluminum oxide (AAO) film. The AAO film was attached to the GE by the radio frequency magnetron sputtering method. The deposition of AuNPs was electrochemically deposited by applying a constant voltage. The GE/AAO/AuNPs were then incubated in an ssDNA probe to capture the target DNA specifically. The hybridization process was performed at 50°C [39]. In situ fabrication of SWCNTs and maintaining the surface area of AAO on the electrode are somehow difficult. In other work, the use of fluorine-doped tin oxide glass electrode (FTO) as the working electrode has been reported by Narang et al. (2016). Nanocrystal zeolite (Nanocrys Zeo) was electrochemically deposited onto the FTO. Unlike other immobilization processes, in this work, the ssDNA was electrochemically deposited by applying a voltage (DPV mode) to get FTO/Nanocrys Zeo/ssDNA. Interestingly, the hybridization process was also performed by applying voltage without heating [40].

After the hybridization process, all HBV genosensors mentioned above were measured by EIS. The hybridization of ssDNA with target DNA on the electrode surface generally hinders the electron transfer. Therefore, *R*_ct_ value represents a number of dsDNA copies attached to the working electrode. From the impedance spectra obtained from those methods, the *R*_ct_ values were increased as the number of target DNA increased. It showed that this *R*_ct_ value agreed well with the amount of HBV DNA. The analytical performances of each method are listed in Table 5.

#### 2.7.2. Photoelectrochemical and Electrochemiluminescence

Photoelectrochemical detection is based on the photocurrent generated from a light source. A photoelectrochemical immunosensor was fabricated by drop-casting gold nanoparticles/ZnAgInS quantum dot (AuNPs/ZnAgInS QDs) nanocomposites on the GCE surface. Subsequently, anti-HBsAg was cross-linked on the AuNPs/ZnAgInS/GCE. Anti-HBsAg/AuNPs/ZnAgInS/GCE was incubated in HBsAg prior detection. White light LED was used as the excitation light source, the photocurrent signal was recorded. As the HBsAg concentration increased, the photocurrent signal decreased because the anti-HBsAg active sites were more occupied by HBsAg, causing inefficient electron transfer. The linearity range and detection limit of the photochemical immunosensor for HBsAg were 0.005–30 ng·mL^−1^ and 0.5 pg·mL^−1^ [41].

Electrochemiluminescence (ECL) transduction technique is based on the electron transfer reactions that induce emitting light on the electrode. This technique is mostly selected due to no spectral interference from the light source. A sandwich ECL HBV DNA sensor was assembled by dropping CdTe QD conjugated capture DNA on GCE modified with graphene nanosheet labeled as CdTe QDs-capture DNA/GNs/GCE. The hybridization of target DNA with the obtained CdTe QDs-capture DNA/GNs/GCE was done at 90°C. After cooling to room temperature, AuNPs-probe DNA was annealed to target DNA/CdTe QDs-capture DNA/GNs/GCE at 90°C as well. The ECL signal obtained from the prepared AuNPs-probe DNA/target DNA/CdTe QDs-capture DNA/GNs/GCE with the help of a three-electrode system was recorded at 555 nm. The linearity range and detection limit of the ECL sensor for HBV DNA detection were 0.0005–0.5 nM and 0.082 pM, respectively [42, 43].

## 3. Paper-Based Analytical Devices for Detection of Hepatitis B

Miniaturization of detection devices to achieve point-of-care testing (POCT) has been developed in the last few decades. Microfluidic paper-based analytical device (*µ*PAD) is one of the most promising platforms for miniaturizing bulky instruments to achieve POCT devices. All the transducer and analytical performances of each *µ*PAD are listed in Table 6. It is mainly owing to its physical properties, which are lightweight, biodegradable, disposable, flexible, and cheap. An immunochromatographic assay (ICA) sandwich immunoassay for HBsAg detection has been proposed for the detection by naked eyes. Nitrocellulose (NC) membrane was used as the conjugate, sample, and absorbent pad. A monoclonal anti-HBsAg1 (mAb1) and polyclonal anti-HBsAg (pAb) were coated on the NC membrane as a test (*T*) and control (C) lines. Another monoclonal anti-HBsAg2-modified coloring agent, ultramarine blue (mAb2-UMB), was prepared by conjugation carboxyl group of UMB with the amine group of mAb2. Sample containing HBsAg was loaded on the sample pad, and then the sample went through the *T* and C lines by capillary force. As the HBsAg was present, mAb1 (T line) captured it, and the remaining HBsAg was captured by pAb (C line). Subsequently, the mAb2-UMB was loaded on the sample pad and bound with HBsAg, the *T* and C lines giving blue colors on both lines. In contrast, blue color on *T* lines would not appear in the absence of HBsAg because capture antibody mAb1 only specifically recognizes HBsAg while pAb on the C line has several epitopes that can bind with mAb2-UMB. To quantify the amount of HBsAg, blue intensity on the *T* and C lines was measured using ImageJ software [44].

An AuNP-based lateral flow assay (LFA) for HBV DNA colorimetric detection also has been proposed by Choi et al. (2016). The device was made of 4 integrated layers with PVC as the backing pad; (1) lateral flow strip with AuNP-capture DNA (AuNPs-cDNA) immobilized on sample pad, shunt and polydimethylsiloxane (PDMS) droplets, *T* and C lines, and absorption pad; (2) glass fiber for DNA amplification; (3) fast technology analysis (FTA) card; (4) cellulose for absorption pad of waste. The whole blood sample was loaded on the third layer, the FTA card, to extract nucleic acid at room temperature, and then the waste was absorbed by the cellulose at the fourth layer. After removing the fourth layer, the amplification reagent was dropped on the second layer and then mounted with the third layer using disposable tape for amplification at 65°C and denaturation at 95°C. It was then combined with the first layer to allow the extracted target DNA to hybridize with AuNPs-cDNA on the first layer. The AuNPs-cDNA-target DNA was moved laterally through the NC membrane until reaching C line. The intensity was measured to quantify the amount of HBV DNA. The use of shunt and PDMS on the top layer helped in increasing the sensitivity of the proposed assay [45].

Electrochemical detection has been frequently used as the signal transducer because it provides a good selectivity and a wider linearity range. By combining the advantages of PADs and electrochemical detection, electrochemical paper-based analytical devices (ePADs) have been developed. An ePAD has been reported by Chen et al. in 2018. It employed the electrochemiluminescence (ECL) detection method. The device consists of two parts, a screen-printed electrode (SPE) and a paper-based biosensing platform. The paper was cut to get the same size as the SPE. It is known that most of the biomolecules have no optical properties, especially when the detection method used was ECL. Thus, the HBsAg sample needed to be labeled with sulfonated magnetic beads coated Ab_1_ and Ru(bpy)_3_^2+^-NHS labeled Ab_2_ as ECL probe prior analysis. The ECL signal was measured immediately after dropping the sample solution on the paper using a custom portable photomultiplier [46]. The paper-based device and the SPE needed to be assembled before analysis, making it less simple.

Recently, there have been developed paper folding or 3D ePADs to detect DNA amplification of HBV for early diagnosis of hepatitis B. The 3D conformation makes the possibility of a single device to be able for sample preparation and detection process simultaneously. In 2015, the 3D ePAD was designed to be foldable and had a moveable separator to create a hollow in order to separate the sample solution with the printed electrodes, so-called slipPAD. The design of slipPAD allowed the sample to be treated before detection. The slipPAD has a separator paper to allow the solution to be incubated and reacted with an oxidant, MnO_4_^−^, before detection. The detection principle of the proposed device was using metalloimmunoassay, which is a kind of hepatitis B labeled DNA with silver nanoparticles (AgNPs) and magnetic microbeads (M*µ*B) as the solid-phase support. As aforementioned, the slipPAD has a hollow channel for the incubation step, and the MµB was used as the solid-phase support in the washing step and as the signal amplification material. The created hollow can also be used as the preconcentration step because the DNA was labeled with MµB making it bigger in size. After the preconcentration step was done, the separator paper was slipped to allow the preconcentrated labeled DNA with AgNPs to oxidize to Ag^+^, leading to contact with the working electrode. The free Ag^+^ ions in the solution were then detected using anodic stripping voltammetry (ASV) to quantify the amount of hepatitis B labeled DNA in the sample solution [47].

According to the reason mentioned above, the use of labeled HBsAg to determine the concentration of HBsAg is not preferable. Label-free ePAD had also been proposed by Srisomwat et al. in 2020. The configuration of the device was like a pop-up paper, able to open and close. The aims of the pop-up ePAD are still to make a compact device that can accommodate sample preparation and detection steps by simple folding. A pyrrolidinyl peptide nucleic acid, acpcPNA, was covalently bonded with the cellulose paper on the backside of the device. The incubation step was performed to allow acpcPNA hybridized with HBV DNA in an open paper configuration. Once the incubation step was done, the paper was folded to be in close paper configuration. A reagent, [Fe (CN_6_)^3−/2−^], was introduced into the reagent zone allowing the reactant to react with incubated HBV DNA, and the signal was recorded using DPV [48]. The pop-up ePAD for HBV DNA detection has demonstrated the potential to be POCT devices, except accessibility. Recent work has shown easy accessibility to a smartphone-based device.

## 4. Conclusions and Outlook

As conclusions, the development of analytical devices for the detection of hepatitis B virus has been developed rapidly in the last two decades. Conventional PCR has been tremendously used as the standard method to quantify HBV DNA. PCR has shown great accuracy, yet it needs proper instruments and operators, which are difficult to carry in rural areas. Over the past few years, HBV DNA and HBsAg are the most often used biomarkers to determine the amount of hepatitis B virus presence in fluid body samples. Colorimetric ELISA-based immunosensors are the most convenient recognition mechanism to detect HBsAg due to specific interaction between HBsAg and enzyme-linked anti-HBsAg. To increase the specificity, there has been developed sandwich ELISA-based immunosensors to double-check a specific antigen. As a consequence, labeling antigen with another enzyme-linked anti-HBsAg (different epitope) is necessary. The sandwich ELISA-based immunosensors are promisingly able to enhance the sensitivity if the labeled antigen has several enzyme-probes. The HBV DNA biosensors have a similar sensing mechanism as direct and sandwich recognition mechanisms. However, labeling biomarkers is a complicated and time-consuming process and requires high-cost reagents.

Electrochemical detections for quantifying HBV biomarkers are the most versatile transducers ranging from CV, SWV, and DPV until EIS. Researchers somehow still need to label the biomarkers to recognize the HBV biomarkers specifically. Since biomolecules are generally not electrochemically active, modifying working electrodes to increase the electron transfer is necessary. Nanoparticles, composites, and physically and chemically coated-film are commonly used to enhance sensitivity.

Nowadays, the miniaturization of analytical devices is a new challenging work. The most recent platform is microfluidic paper-based analytical devices (*µ*PADs). *µ*PADs with various detection systems have been developed depending on the properties of the analyte. The advantages of *µ*PADs over other analytical devices are flexible, biodegradable, and easy to use. In addition, the integration of *µ*PADs with a smartphone has also been fascinating to develop. This technique will lead to point-of-care testing (POCT), which will be very useful to use as an alternative device to detect biomolecules such as viruses and bacteria in general. Hence, there is still a big room to develop a rapid, sensitive, label-free, portable, disposable, and biodegradable device.

## Figures and Tables

**Table 1:** ELISA-based colorimetric detection of HBV.

Biomarker	Recognition method	Linearity range	Detection limit	Ref.
HBsAg	ELISA	0–40 ng·mL^−1^	1 ng·mL^−1^	[6]
HBsAg	ELISA	NS^*∗*^	NS^*∗*^	[17]

^*∗*^NS, not specified due to qualitative analysis.

**Table 2:** Fluorescence detection of HBV.

Biomarker	Fluorescent probe	Linearity range	Detection limit	Ref.
HBV DNA	AgNP aggregates	100 fM–10 nM	50 fM	[19]
HBsAg	Carboxyl-modified quantum dot beads (QBs)	75 pg·mL^−1^–4.8 ng·mL^−1^	75 pg·mL^−1^	[6]
HBsAg	Mercaptopropionic acid-modified CdTe quantum dots (MPA-QDs)	47–380 pg·mL^−1^ and 0.75–12.12 ng·mL^−1^	1.16 pg·mL^−1^	[21]
HBsAg	Modified europium nanoparticles	0.01–100 ng·mL^−1^	0.25 ng·mL^−1^	[20]
mutDNA	SYBR green I	0.1–40 nM	0.05 nM	[7]

**Table 3:** Raman spectroscopy detection of HBV.

Biomarker	Recognition method	Linearity range	Detection limit	Ref.
HBV DNA	PCR-Raman	NS^*∗*^	NS^*∗*^	[8]
HBsAg	SERS-active	0.00125–60 IU/mL	0.01 IU/mL	[23]
HBsAg	LSPR	100 fg·mL^−1^–10 ng·mL^−1^	100 fg·mL^−1^	[24]

^*∗*^NS, not specified due to qualitative analysis.

**Table 4:** Electrochemical detection of HBV.

Biomarker	Linearity range	Detection limit	Ref.
*Potentiometry*			
HBsAg	4–960 ng·mL^−1^	1.9 ng·mL^−1^	[10]
	0.01 ng·mL^−1^	0.08–10 ng·mL^−1^	[25]

*Amperometry*			
HBsAg	0.0005–10 ng·mL^−1^	166 fg·mL^−1^	[26]
HBeAg	60 fg·mL^−1^−100 ng·mL^−1^	20 fg·mL^−1^	[27]
HBsAg	10–200 *µ*g·mL^−1^	0.17 *µ*g·mL^−1^	[28]

*Voltammetry*			
HBsAg	0.001–0.015 ng·mL^−1^	0.9 pg·mL^−1^	[29]
HBcAg	1.0 to 5.0 ng·mL^−1^	0.89 ng·mL^−1^	[30]
HBsAg	32 fg·mL^−1^−100 ng·mL^−1^	10.2 fg·mL^−1^	[31]
HBsAg	0.1–300 pg·mL^−1^ and 0.1 to 1,000 pg·mL^−1^	60 fg·mL^−1^ and 10 fg·mL^−1^	[32]
HBsAg	20 fg·mL^−1^−200 ng·mL^−1^	6.7 fg·mL^−1^	[33]
HBV DNA	1.10 × 10^3^–1.21 × 10^5^ copies mL^−1^	1100 copies mL^−1^	[2]
HBV DNA	0.18 × 10^−6^−1.8 × 10^−6^ M	2.61 nM	[34]

**Table 5:** EIS detection of HBV.

Biomarker	Linearity range	Detection limit	Ref.
HBV DNA	1.0 × 10^−18^–1.0 × 10^−6^ M	1.0 × 10^−6^ M	[9]
HBV DNA	150–10^6^ copies mL^−1^	50 copies mL^−1^	[40]
HBV DNA	10^2^–10^3^ and 10^3^–10^5.1^ copies mL^−1^	111 copies mL^−1^	[39]

**Table 6:** Paper-based analytical devices (PADs) with various detections for HBV detection.

Biomarker	Detection	Linearity range	Detection limit	Ref.
HBsAg	ECL	34.2 pg·mL^−1^ to 34.2 ng·mL^−1^	34.2 pg·mL^−1^	[46]
HBsAg	Colorimetric	1–50 ng·mL^−1^	0.37 ng·mL^−1^	[44]
HBV DNA	Colorimetric	NS^*∗*^	100 IU·mL^−1^	[45]
HBV DNA	ASV	0–500 pM and 0–1.5 nM	85 pM	[47]
HBV DNA	DPV	50–100 pM	1.45 pM	[48]

^*∗*^NS, not specified due to qualitative analysis.

## Data Availability

The data used to support the findings of this study are included in the article.
